# CeO_2_ facets control: from single (100) to multiple†

**DOI:** 10.1039/c9ra09731g

**Published:** 2020-01-07

**Authors:** Wenzhe Si, Yu Wang, Yue Peng, Jianjun Chen, Junhua Li

**Affiliations:** State Key Joint Laboratory of Environment Simulation and Pollution Control, National Engineering Laboratory for Multi Flue Gas Pollution Control Technology and Equipment, School of Environment, Tsinghua University Beijing 100084 P. R. China chenjianjun@tsinghua.edu.cn; Department of Chemistry, Carnegie Mellon University Pittsburgh PA 15213 USA; School of Environmental Science and Engineering, Yancheng Institute of Technology Yancheng 224051 P. R. China

## Abstract

The synthesis of high energy facets and tunable low energy facets was achieved in CeO_2_ films on a SrTiO_3_ substrate by pulsed laser deposition. Three different facets of CeO_2_ with distinctive morphologies appeared consecutively. Firstly, the (100) facet of CeO_2_, which is the highest energy surface, was grown on the SrTiO_3_ (STO) (100) substrate. The surface energy was decreased gradually with the increase of the laser pulse shots, and the (110) and (111) LEF appeared consecutively. The three different facets presented distinctive morphologies and the interface between each facet could be easily observed by the cross section of Transmission Electron Microscope (TEM) imaging. The interface between the (100) and (110) facet may exhibit excellent oxygen storage capacity, even better than the (100) facet.

## Abstract 2

Three different facets of CeO_2_ films with distinctive morphologies could be synthesized and tuned on a SrTiO_3_ substrate by pulsed laser deposition.

## Graphical abstract

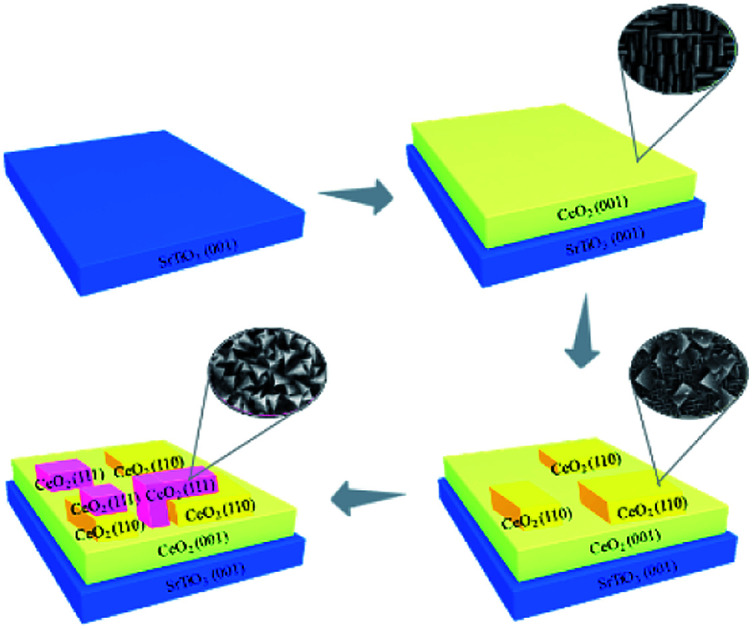

## 1. Introduction

Oxide facets with distinctive crystallographic features possess varied atomic terminating characters, which are found to exhibit significant differences in magnetism, sensing and especially catalysis.^1–3^ Rational design and orientable control of crystal facets are commonly adopted to optimize their performances.^4–7^ High energy facets (HEF) that have abundant unsaturated active sites usually exhibit excellent properties.^8,9^ But they are often unstable, and difficult to obtain by traditional chemical methods.^10–12^ The exploration of advanced synthetic approaches to oxides with well-defined HEF is in urgent demand and of great importance.

In addition, some particular combinations of HEF and low energy facets (LEF) have been proven to show outstanding performances, even better than HEF.^1,3^ Yu *et al.* found that the combination of TiO_2_ anatase (001) (*ca.* 70%) and (101) (*ca.* 30%) facets was more active than the clean (001) which is the traditional highest energy surface of TiO_2_.^3^ Pan *et al.* synthesized (001) and (230) facets of BaTiO_3_ sensitized with CdSe quantum dots, which exhibited better photocatalytic activity than (001) facet.^1^ The superior activities were due to the charge separation and electronic reduction activities resulting from the synergy effect of (001) and (230) facets. However, controllable synthesis of the oxides with dominant HEF and tunable LEF is still a great challenge.

**Scheme 1:**
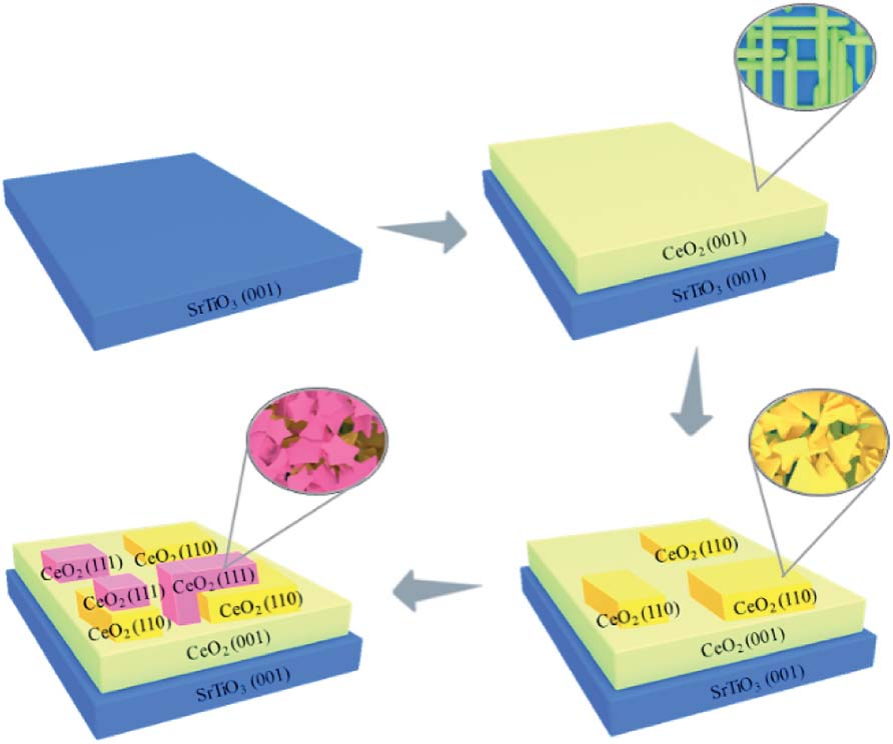
Synthesis route of the CeO_2_ films on SrTiO_3_ substrate.

Here a facile method to controllably synthesize and tune the oxides facets by pulsed laser deposition (PLD) was reported (Scheme 1). CeO_2_, which is an important material utilizing in fuel solar cells, gates for metal–oxide semiconductor devices, phosphors and three-way catalysts, was selected as a target.^13–18^ Firstly, (100) facet of CeO_2_, which is the highest energy surface, was grown on the SrTiO_3_ (STO) (100) substrate.^19,20^ The high laser power induced CeO_2_ to grow epitaxially along (100) facet. The surface energy was decreased gradually with the increase of the laser pulse shots, and (110) and (111) LEF appeared consecutively. The three different facets presented distinctive morphologies and the interface between each facet could be easily observed by the cross section of Transmission Electron Microscope (TEM) imaging. The terminated surface could be tuned by the thickness control by PLD. This method may provide a new sight for the controllable synthesis of the oxides with tunable facets, especially for those oxides with distinctive morphologies on different facets.

## 2. Materials and methods

### 2.1 Chemicals

CeO_2_ powder was purchased from Shanghai Yuelong Company of analytical grade purity and used without any further purification. STO (100), STO (110) and Si (100) substrates were purchased from Hefei Kejing Materials Technology Company, LTD. The CeO_2_ powder was finely grinded and then pressed into a pellet (diameter is 2 cm, and thickness is 0.8 cm). Afterwards, the resulting pellet was calcined to target at 1000 K for 10 h in the air.

### 2.2 Preparation of CeO_2_ films

The CeO_2_ films can be achieved on STO (100), STO (110) and Si (100) substrates by the PLD method using a KrF excimer laser with a wavelength of 248 nm, a laser fluence of 1.5 J cm^−2^ and a repetition rate of 3 Hz. The distance between a ceramic target and a substrate is 8 cm. Here the CeO_2_ films were deposited on STO (100) for different pulse shots: 1000, 5000, 10 000, and 50 000, respectively. STO (110) and Si (100) for 5000 shots. The temperatures of films growing are 800 K. Then the films were annealed maintained at the growth temperature for 0.5 h. The temperature was subsequently lowered at a rate of 1 K min^−1^. The films with different deposition pulse shots for 1 000, 5 000, 10 000, and 50 000 are abbreviated as S_1_, S_5_, S_10_, and S_50_, respectively.

### 2.3 Characterization

The crystal structure of the CeO_2_ films were identified by X-ray diffraction (XRD, Rigaku D/MAX 2500 V/PC) with Cu Kα radiation (*λ* = 1.5418 Å) at 40 kV and 30 mA at room temperature.

Scanning electron microscopy (SEM) images were acquired using a focused ion beam (Helios Nanolab 600i). Since the CeO_2_ films were poorly conductive, the conductive adhesive was used to fix the film on the specimen holder and avoid charge accumulation when ion beam irradiation.

The surface morphology was measured by an atomic force microscopy (AFM, SPI 3800 N Probe Station + SPA 400 unit system), which was manufactured by SII NanoTechnology Inc. DFM (Dynamic Force Mode) scanning mode was selected in the experiments mentioned above, while the vibration frequency of cantilevers is about 150 KHz.

Transmission electron microscopy (TEM) was carried out using a JEM-2100F at 200 kV. X-ray photoemission spectroscopy (XPS, Thermo ESCAlab 250) experiment was performed using monochromatic Al Kα radiation (1486.6 eV). TEM specimens were extracted from the cross-section of CeO_2_ film by the FIB lift-out technique. To prevent damage to the film from focused Ga^3+^ ion beam, W(CO)_6_ gas [W(CO)_6_(EBD)] was injected to the interested region with electron beam deposition to form a thin protective layer of tungsten composite. After W(CO)_6_(EBD), focused ion beam deposition was continued in case of damage from milling process. The lift-out specimen was mounted onto a crescent copper grid with FIB deposition of W(CO)_6_ [W(CO)_6_(IBD)] and then mill it to the right thickness. After a low voltage bombard process, amorphous layer, which formed during the milling process, were removed and the specimen was transferred to TEM characterization.

X-ray photoelectron spectroscopic (XPS) was used with Mg Kα (*hν* = 1253.6 eV) X-ray source to determine the Ce 3d binding energies (BEs) of surface cerium species. The C 1s signal (BE = 284.6 eV) of contaminant carbon was employed to calibrate the BE values of Ce 3d.

## 3. Results and discussion

### 3.1 XRD results

**Fig. 1:**
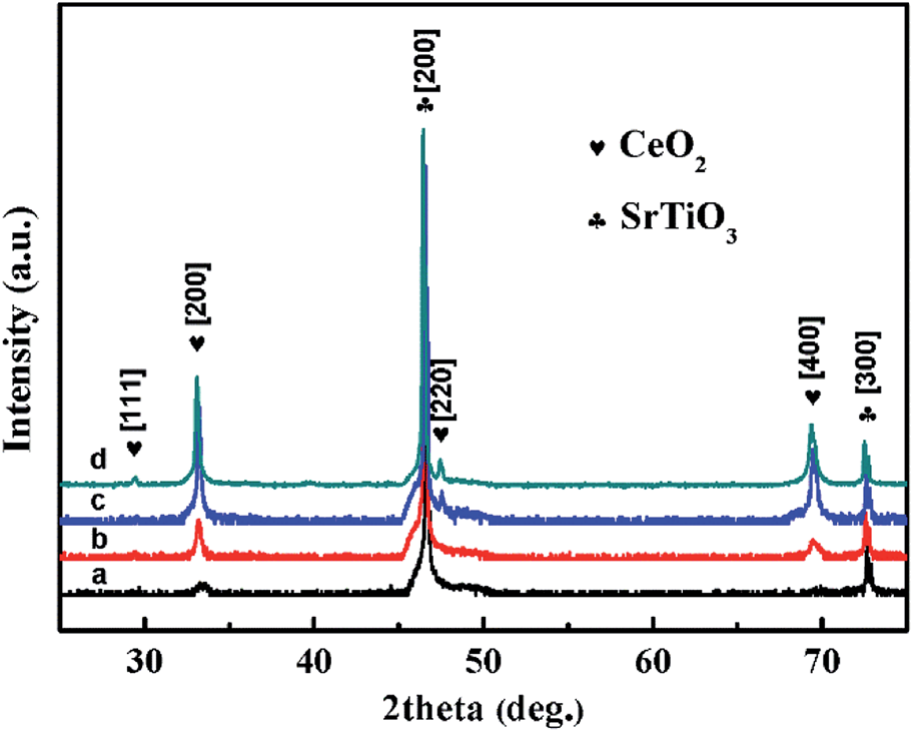
XRD patterns of CeO_2_ films which were labeled as (a, b, c, and d) for sample S_1_, S_5_, S_10_ and S_50_, respectively.

The X-ray diffraction (XRD) patterns of CeO_2_ films with different pulse shots deposited on STO (100) substrates are shown in Fig. 1. All the peaks have been normalized. For the S_1_ sample, only (200) plane of CeO_2_ cubic fluorite phase can be observed. When the pulse shots increased up to 5000, (400) plane appeared and the peak intensity of (200) was enhanced. Based on the results of S_1_ and S_5_, which represent the initial growing stage, the CeO_2_ film has a preferred orientation along the (100) direction. For the S_10_ sample, (200) and (400) planes of CeO_2_ still existed and were enhanced in intensity, compared to S_1_ and S_5_. In the meanwhile, a new peak around 47° appeared. This can be indexed as the (220) plane. The results indicated that the facets of CeO_2_ film varied from single (100) to both (100) and (110) by increasing the pulse shots. For the S_50_ sample, a new direction peak due to CeO_2_ (111) plane was obtained. The coexistence of (100), (110) and (111) occurred in CeO_2_ films, which may be responsible for the surface energy variation. From the appearances of LEF, it indicated that the surface energies of the CeO_2_ films decreased with increasing the film thickness.

### 3.2 Morphologies

**Fig. 2:**
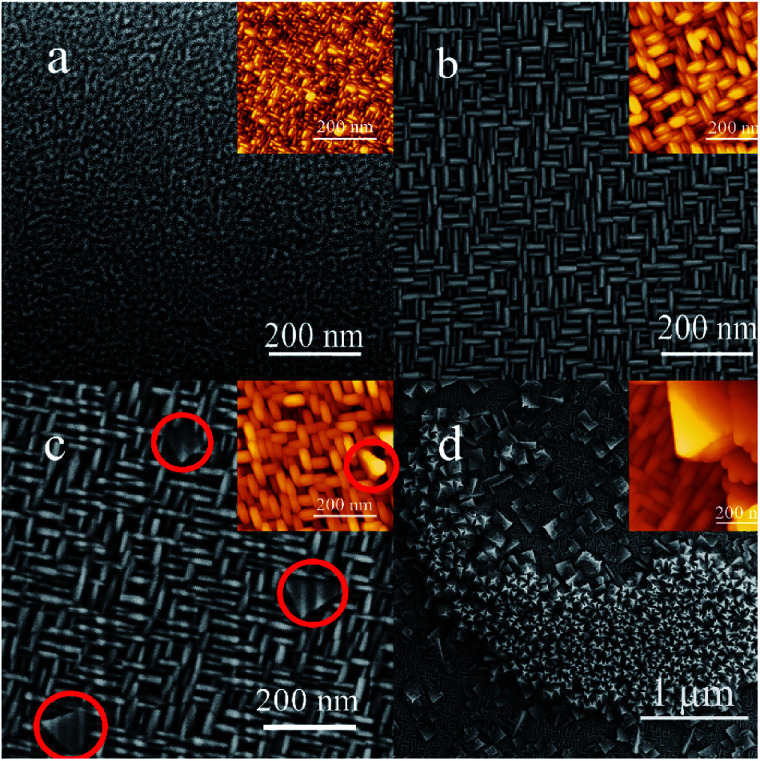
SEM images of CeO_2_ films which are labeled as (a, b, c, and d) for sample S_1_, S_5_, S_10_ and S_50_, respectively; the insets are corresponding AFM images.

Then Scanning Electron Microscope (SEM) and Atomic Force Microscope (AFM) were carried out to show the morphologies of these three different facets, and detect the surface where (110) and (111) planes grow on (Fig. 2). The conductive adhesive was used when the SEM images were acquired. The role of conductive adhesive is usually divided into two parts: one role is to avoid charge accumulation. It is a common approach to improve the SEM resolution. Another role is to fasten the sample. It can be seen that the shape of S_1_ was 3D linen-like nanorods. However, its morphology did not show good uniformity. For the S_5_ sample, the grid structure became more regular (Fig. 2b). The nanorods with two different directions were almost equal in length and diameter, and the size of the pores (20–50 nm) was stacked by the nanorods, which may create large surface area in the framework. For Fig. 2c, a few nanosheets (shown in the red circles) with triangle form grew on top of the nanorods. Correlating with the XRD results that (100) and (110) facets coexisted for S_10_, it can be inferred that the triangle-like nanosheets with (110) facet was grown up from the nanorods, which could be attributed to the decrease of surface energy. For the S_50_ sample (Fig. 2d), new structure consisted of the star-like nanosheets appeared. What's more, single (110) and (111) facets of CeO_2_ films were synthesized separately on STO (211) and Si (001) substrates (Fig. S3 and S4†). It can be seen that the morphologies were corresponding to what we obtained on STO (001) by tuning the thickness of films. The results suggested that three different structures with distinctive morphologies could be formed on S_50_ sample simultaneously, and each structure was oriented along to the different directions.

### 3.3 TEM results

**Fig. 3:**
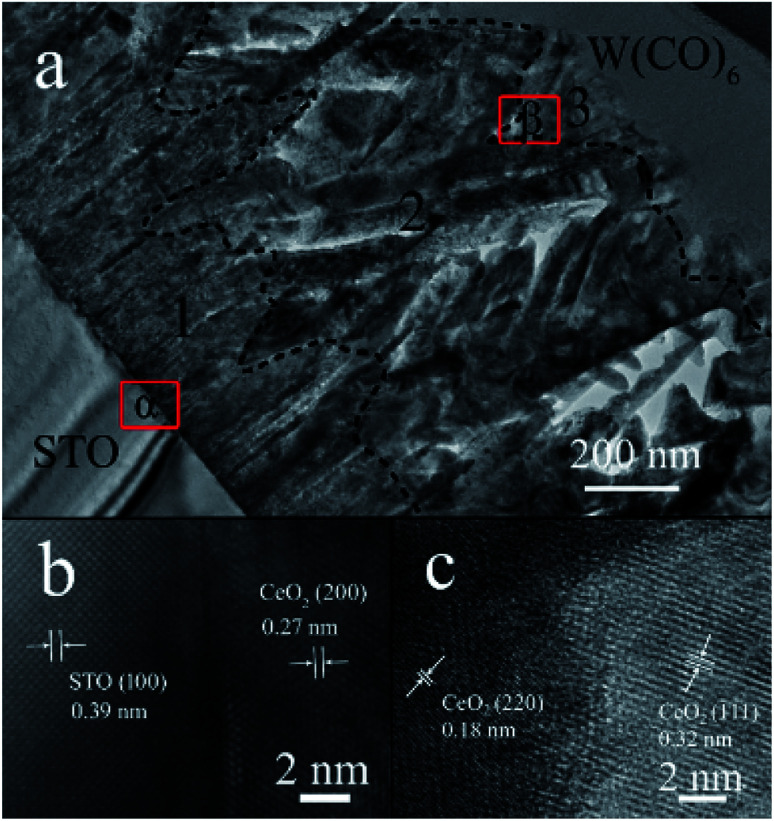
(a) TEM characterization of cross section of S_50_. (b and c) HRTEM of region α and β in (a), respectively.

For local crystal lattice determination, a small cross section of S_50_ was prepared by Focused Ion Beam (FIB) for TEM imaging. Different morphologies of CeO_2_ facets and the transformation position of each structure can be easily observed from Fig. 3a. The region 1, 2 and 3 represented the linen-like nanorods, triangle-like nanosheets and star-liked nanosheets, respectively. Fig. 3b showed the interface between CeO_2_ (100) facet and STO (001) substrate. The lattice spacing was 0.27 nm, according with the (200) lattice of CeO_2_ cubic phase. It could be further confirmed that the nanorods oriented along (100) facet. Fig. 3c presented the HRTEM of region β, and it can be observed that lattice distortion occurred between (110) and (111) facets, which might be responsible for the stress function among the interfaces. According to the cross section of TEM imaging, the starting position of each facet could be easily confirmed, which help to tune the terminated surface of the film by controlling the thickness of the film.

### 3.4 XPS results

**Fig. 4:**
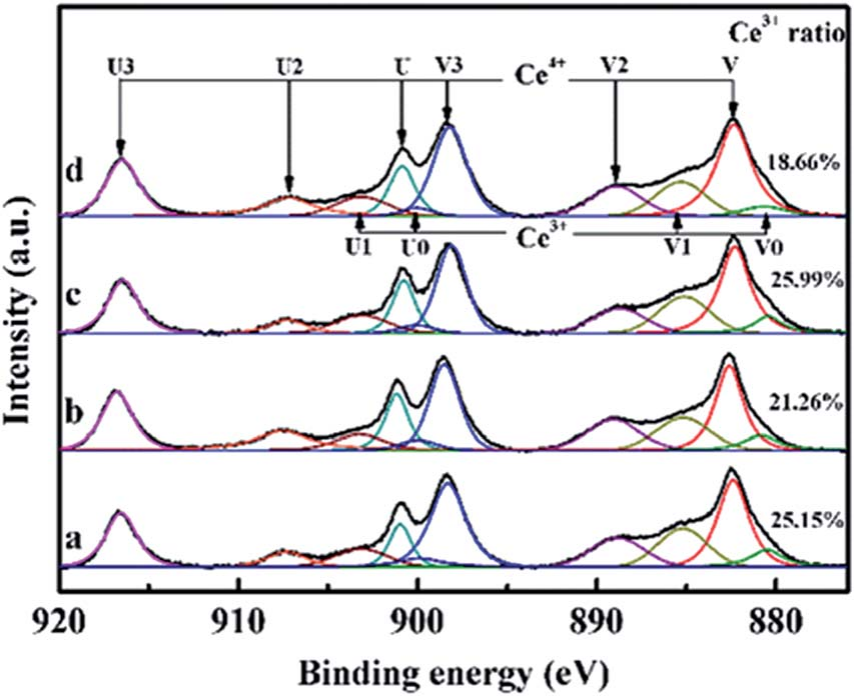
Ce 3d XPS spectra of CeO_2_ films which are labeled as (a, b, c, and d) for sample S_1_, S_5_, S_10_ and S_50_, respectively.

Ce 3d X-ray Photoelectron Spectroscopy (XPS) spectra of CeO_2_ films with different pulse shots are shown in Fig. 4. The peaks labeled U are due to 3d_3/2_ spin–orbit states, and those labeled V are the corresponding 3d_5/2_ states. The U3/V3 doublet is due to the primary photoemission from Ce^4+^–O_2_. The U/V and U′/V′ doublets are shakedown features resulting from the transfer of one or two electrons from a filled O 2p orbital to an empty Ce 4f orbital. The U1/V1 doublet is due to photoemission from Ce^3+^ cations. This shakedown feature gives rise to an additional doublet, which is labeled as U0/V0 in the spectrum. Ce^3+^ ratio showed a downward trend by increasing the pulse shots, except for S_10_. The oxygen storage capacity of CeO_2_ mainly relies on the intrinsic oxygen vacancies of the surface, which is attributed to the rapid redox cycles of Ce^4+^ ↔ Ce^3+^.^21,22^ In CeO_2_ structure, oxygen storage capacity is related with the Ce^3+^/(Ce^3+^ + Ce^4+^). The more the amount of Ce^3+^ exists, the more the oxygen vacancies exists. The material can adsorb more oxygen with the increase of the amount of oxygen vacancy. As the interface between the (100) and (110) facets possessed the largest the amount of Ce^3+^ ratio (25.99%), it is indicated that it may possess the highest oxygen storage capacity.

## 4. Conclusions

In summary, a facile PLD method was presented for the synthesis and tuning of the exposed facets of CeO_2_ films. First, (100) HEF of CeO_2_ was grown on the STO (100) substrate. With increasing the pulse shots, (110) and (111) LEF appeared consecutively resulting from the decrease of surface energies. Each facet had distinctive morphologies and the positions of the interfaces between each facet can be directly observed from the cross-section of the CeO_2_ film by TEM. The interface between (100) and (110) facet showed highest Ce^3+^ ratio, which led to the excellent oxygen storage capacity. This work provides a simple way to tune CeO_2_ film from single HEF to multiple, and shows potential possibility to controllably synthesize the oxides with dominant HEF and tunable LEF.

## Conflicts of interest

There are no conflicts to declare.

## Supplementary Material

RA-010-C9RA09731G-s001
