# Supplementary material for: CeO2 facets control: from single (100) to multiple
Source: RSC Adv. 2020 Jan 7;10(3):1271–4. doi: 10.1039/c9ra09731g (PMC9048269; doi:10.1039/c9ra09731g)
Supplement: RA-010-C9RA09731G-s001 [file RA-010-C9RA09731G-s001.pdf]

Electronic Supplementary Material (ESI) for RSC Advances.
This journal is © The Royal Society of Chemistry 2019

Supporting Information

CeO_2 facets control: from single (100) to multiple

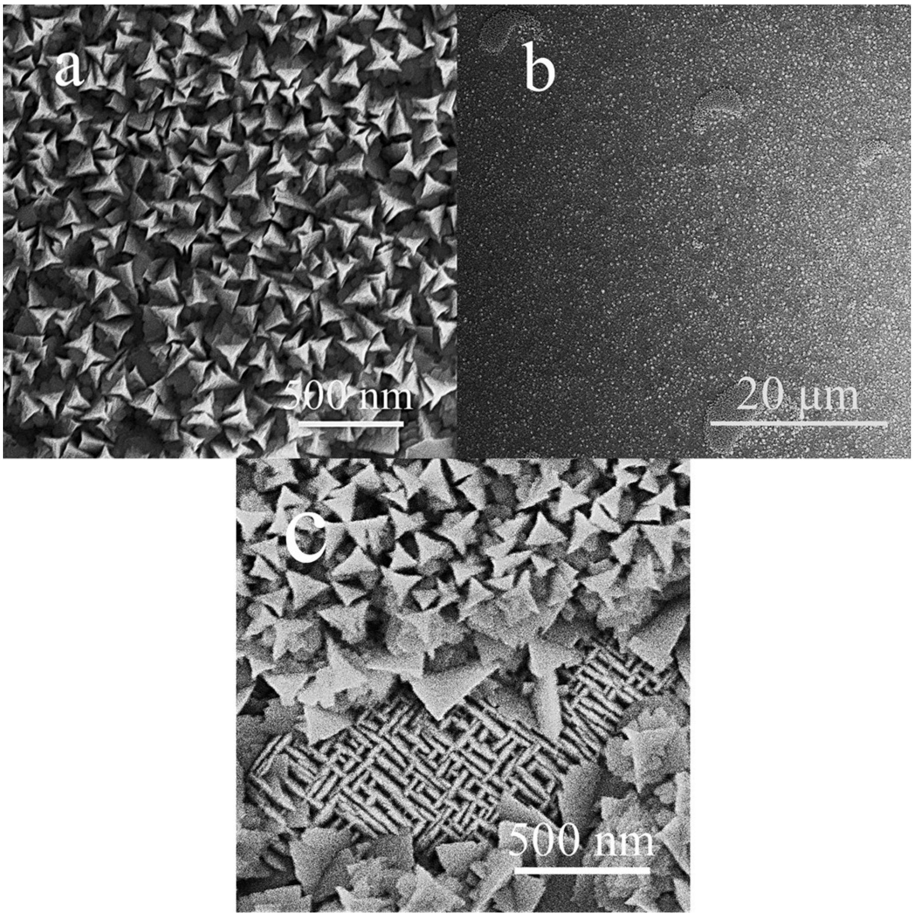

Figure S1. (a) SEM images of star-like nanosheets mentioned in Figure 2d. (b, c) SEM images of S_{50}.

Figure S1 shows the SEM images of S_{50}. From figure S1(c) it can be seen that the star-like nanosheets were grown on the top of the triangle-like nanosheets. No star-like nanosheets existed on the top of the nanorods. It can be further demonstrated that the (111) facet of CeO_2 grown on the top of (110) facet, not the (100) facet or the substrate.

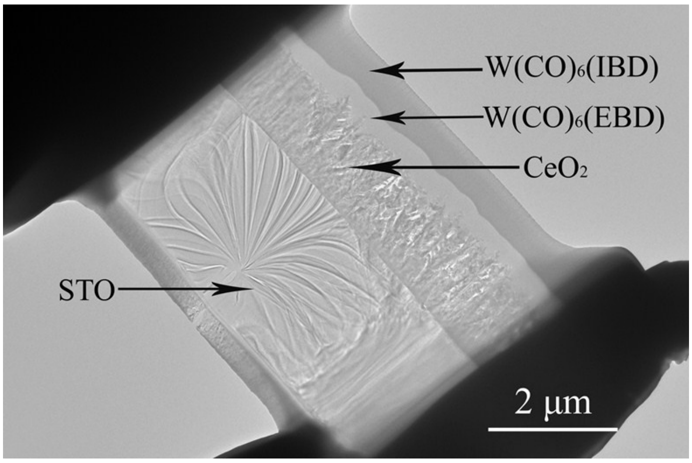

Figure S2. SEM images of cross-section of S_{50}.

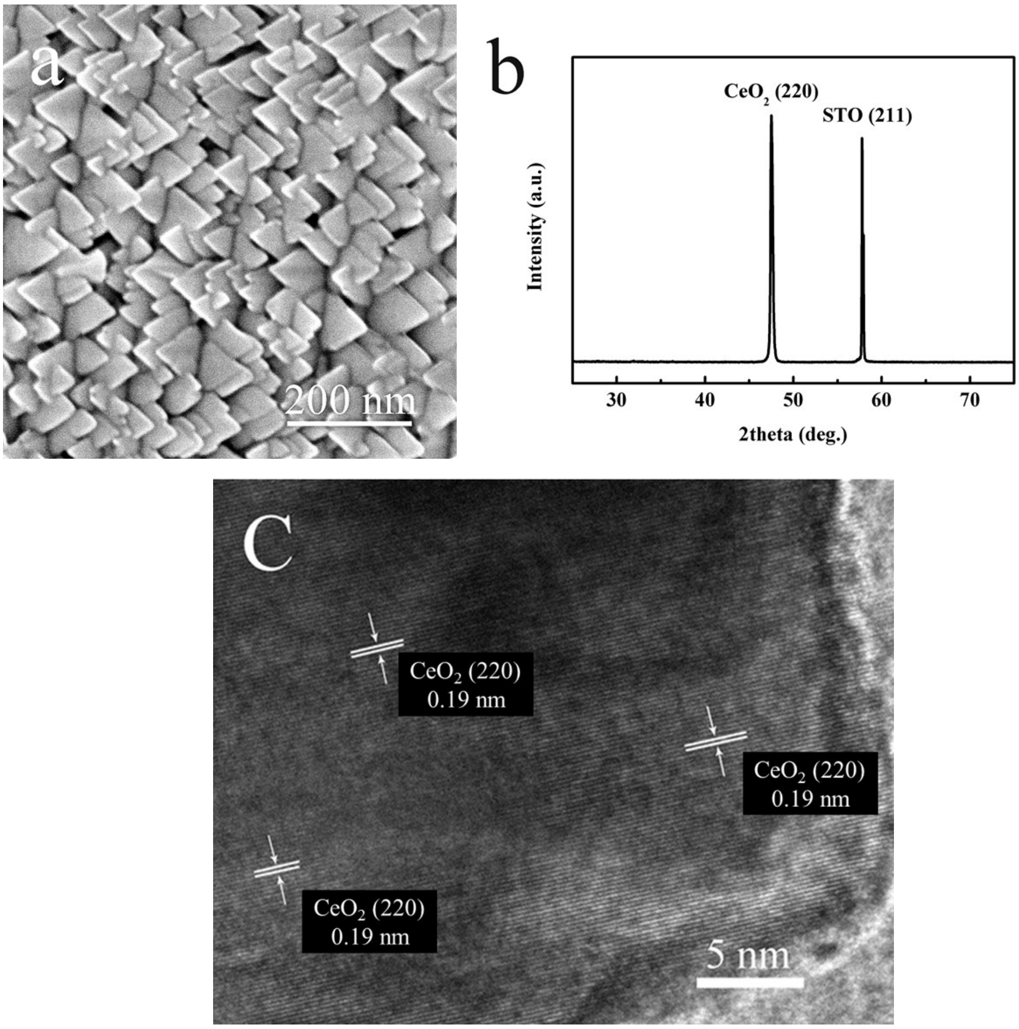

Figure S3. (a) SEM image of CeO_2 film on STO (211) substrate. (b) XRD pattern of CeO_2 film on STO (211) substrate. (c) HRTEM image of CeO_2 film on STO (211) substrate.

The CeO_2 film on STO (211) was prepared to confirm the terminated facet of the triangle-like nanosheets. From the SEM image (figure S3(a)), it is shown that the morphology is just like triangle-like nanosheets. According to the XRD pattern (figure S3(b)) and HRTEM (figure S3(c)), it can be confirmed that the orientation is along (110) facet.

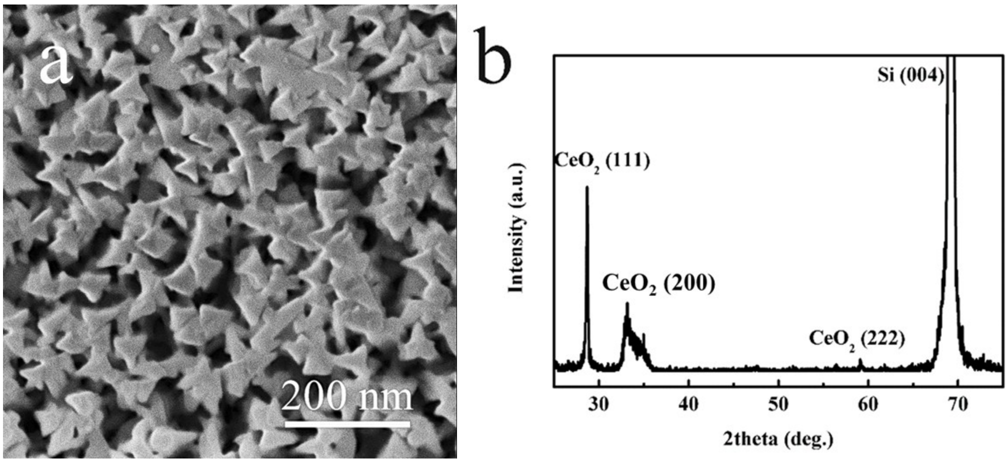

Figure S4. (a) SEM and (b) XRD of CeO_2 film on Si (001) substrate.

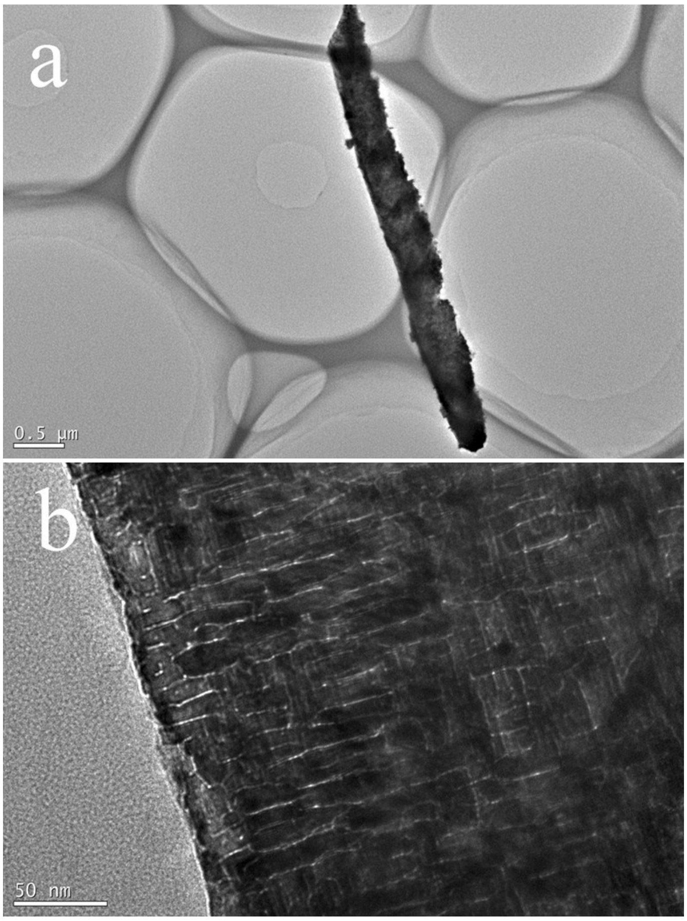

Figure S5. (a, b) TEM images of CeO_2 nanorods took from sample S_5.
